# 

**Published:** 1861-10

**Authors:** 


					﻿,	Published Monthly, at $3 per Annum in Advance.	j
!■ Jk. T Z_. T<r T 2k. if
P :	I g
3	' *
. **
ImIIIQI, HII SBIillHI,
. ;	'O'
r
11	I
t	JOURNAL.	1
*	;*	I X
** I	.	S
*	I	I ®
I I	-S'
ai I	S
V ’	-	-	_	5
>, — ----------------------------
S	'9*
.I	■
5 :	M
® :	I
J. G. WESTMORELAND, M. D.,
S Professor of Materia Medica and Medical Jdrisprcdence in the Atlanta Medical College, ■ *
Si	'
© i	EDITOR AND PROPRIETOR.	S
’ ®	2
*"	SI
*	M
® I	2
1
I	4,^:. . - ----------: -	*
« i	Pax et scieiitia, sed verltas sine tiinore.	;
--	----	U,
« ■	®
5	i	J
6	‘	*
« j	  g.
1	Vol. Vn.	“OCTOBER,0'861.'	No. 11. |
« i	s
«•
2	ATLANTA, GA:	J
i	.A.TIL.A.lSrT2L IN-TEUmGrENCKR 3?RI3SrT.	g
«	1861.	I
, *
g_______ ________________ _	_______________;
?	Each Number contains Slxty«Ponr Pagen.	j
				

